# Rapid Purification of Human Bispecific Antibodies via Selective Modulation of Protein A Binding

**DOI:** 10.1038/s41598-017-15748-0

**Published:** 2017-11-14

**Authors:** Adam Zwolak, Catherine N. Leettola, Susan H. Tam, Dennis R. Goulet, Mehabaw G. Derebe, Jose R. Pardinas, Songmao Zheng, Rose Decker, Eva Emmell, Mark L. Chiu

**Affiliations:** 1 Biologics Discovery, Janssen Research & Development, LLC, Spring House, PA 19477 USA; 2 Department of Medicinal Chemistry, University of Washington, Seattle, WA 98195 USA; 3 Biologics Development Sciences, Janssen Research & Development, LLC, Spring House, PA 19477 USA; 4 Biologics Toxicology, Janssen Research & Development, LLC, Spring House, PA 19477 USA

## Abstract

Methods to rapidly generate high quality bispecific antibodies (BsAb) having normal half-lives are critical for therapeutic programs. Here, we identify 3 mutations (T307P, L309Q, and Q311R or “TLQ”) in the Fc region of human IgG1 which disrupt interaction with protein A while enhancing interaction with FcRn. The mutations are shown to incrementally alter the pH at which a mAb elutes from protein A affinity resin. A BsAb comprised of a TLQ mutant and a wild-type IgG1 can be efficiently separated from contaminating parental mAbs by differential protein A elution starting from either a) purified parental mAbs, b) in-supernatant crossed parental mAbs, or c) co-transfected mAbs. We show that the Q311R mutation confers enhanced FcRn interaction *in vitro*, and Abs harboring either the Q311R or TLQ mutations have serum half-lives as long as wild-type human IgG1. The mutant Abs have normal thermal stability and Fcγ receptor interactions. Together, the results lead to a method for high-throughput generation of BsAbs suitable for *in vivo* studies.

## Introduction

Bispecific antibodies (BsAb) show distinct clinical advantages over monoclonal antibodies (mAbs), particularly for dual-antigen targeting, cell redirection efforts, and immune checkpoint modulation and the number of these BsAbs in clinical trials is expanding rapidly^1^. Recent advances in technology to generate BsAbs have led to clinical successes^1–3^. BsAbs can be categorized as containing an Fc region or lacking an Fc region. BsAbs containing an Fc have the advantage of mediating effector functions via interaction with Fcγ receptors, and they generally have long serum half-lives due to their ability to bind the neonatal Fc receptor (FcRn) in a pH-dependent manner.

Screening of large numbers of BsAbs is required to find relevant lead candidates, but the selection process can be challenging due to the need to develop custom purification steps to purify the BsAb from the undesired antibody molecules that can have similar biophysical characteristics as the desired BsAb. These additional steps in purification can lead to either a decrease in yield or purity of the BsAb molecule. Large scale purification of IgG molecules requires robust, preferably single-step purification methods based on commercially available GMP-quality resins such as those based on staphylococcal protein A. Mature protein A contains 5 homologous helical IgG-binding domains, denoted E, D, A, B, and C^4^. Each of these domains is sufficient to bind to the Fc region as well as to the V_H_ region of human V_H_3-family members^5,6^. The crystal structures of either the B domain or a synthetic homolog identified the site of interaction and mutational studies helped elucidate the pH-dependent mechanism of binding between the Fc domain and protein A^7–10^. Stability-enhancing mutations introduced into the B domain, which is the highest affinity Fc binding domain, led to a synthetic fragment termed the Z-domain^11^. Tandem Z-domains have been engineered into commercial Fc affinity resins that are resistant to high pH treatment and which bind only the Fc region^12,13^. Abs are purified using Z-domain affinity resin by binding at neutral pH and eluting in acidic pH buffer.

One method for generating BsAbs, termed controlled Fab-arm exchange (cFAE), involves introduction of a pair of complementary mutations (either F405L or K409R) into the C_H_3 region of two parental mAbs^14^. These sites make important stabilizing intermolecular interactions in human IgG1 and the mutations impart a destabilizing effect on heavy chain homodimers. Co-incubation of parental mAbs harboring these complementary mutations under mild reducing conditions results in >90% heterodimer formation for human IgG1. This cFAE reaction was initially described using purified parental Abs but has recently been shown to be effective when Abs are subjected to cFAE directly from culture supernatants^15^. Although cFAE is relatively efficient for human IgG1 (reactions typically proceed to >90% completion) and can be automated by liquid-handling devices, in supernatant cFAE is desirable for large panels at early stages. The limitation of in supernatant cFAE is the requirement for highly accurate titers of each Ab, which can be laborious or intractable. Additionally, even small amounts of contaminating parental Ab can be harmful when the bivalent molecule has toxic properties. Thus, there is a great need for methods to quickly and reliably produce BsAb, using preferably single-step, universal methods. Previous efforts described introduction of a set of mutations into the CH3 domain to disrupt binding to protein A. When paired with a wild-type parental, the BsAb can be eluted from protein A resin at intermediate pH - allowing separation from parental Abs. Recent work by us and others have addressed improving the efficiency of cFAE in mouse surrogates^16,17^.

Efforts to modulate the protein A binding characteristics of Abs are often associated with significantly decreased serum lifetimes since both protein A and the neonatal Fc receptor (FcRn) share a binding site on the Fc. FcRn is responsible for the transfer of maternal IgG to the fetus and for protecting serum IgG from lysosomal degradation^18,19^. Both of these processes depend on the ability of FcRn to bind with high affinity to IgG at acidic pH (<6.5) in the recycling endosome and to dissociate at neutral pH, releasing the IgG back into the serum^19^. IgG binds FcRn at the C_H_2-C_H_3 interface, such that a single Fc contains two identical FcRn binding sites. Structural and biochemical studies showed that a single Fc binds two FcRn heterodimers, although endocytic trafficking may involve multimerization of FcRn itself on membrane surfaces^20–23^. Several studies have shown that modulating the interaction between the Fc and FcRn strongly impacts serum lifetime^24–29^, leading to the conclusion that FcRn is primarily responsible for determining serum lifetime of IgG in adults. It was shown that mutation of human IgG1 C_H_3 domain residues H435 and Y436 to the IgG3 residues R435 and F436 could abolish binding to protein A and this effect has been exploited for differential protein A-based purification of human IgG1 bsAbs^30^. However, IgG3 isotypes which natively contain R435, have an approximately 3-fold shorter serum half-life compared to IgG1. This short half-life is due to weaker binding of R435-containing molecules to FcRn at acidic pH, leading to increased dissociation from FcRn in recycling endosomes due to competition by IgG1^27,31,32^. Thus, mAbs which contain R435 may have a short half-life compared to their H435 counterparts.

Here, we describe a) the design and preparation of mutations in the C_H_2 domain of the Fc designed to disrupt Z-domain interaction while retaining FcRn binding, b) *in vitro* analysis of the binding between the human IgG mutants and both Z-domain and FcRn, c) development of methods to prepare the BsAbs by differential protein A purification either from controlled Fab arm exchange of purified parental mAbs, culture supernatants; or co-transfection of parental mAbs, and d) pharmacokinetic analysis of the serum lifetime of the mutant BsAbs.

## Results

### Rationale for Mutations

**Figure 1:**
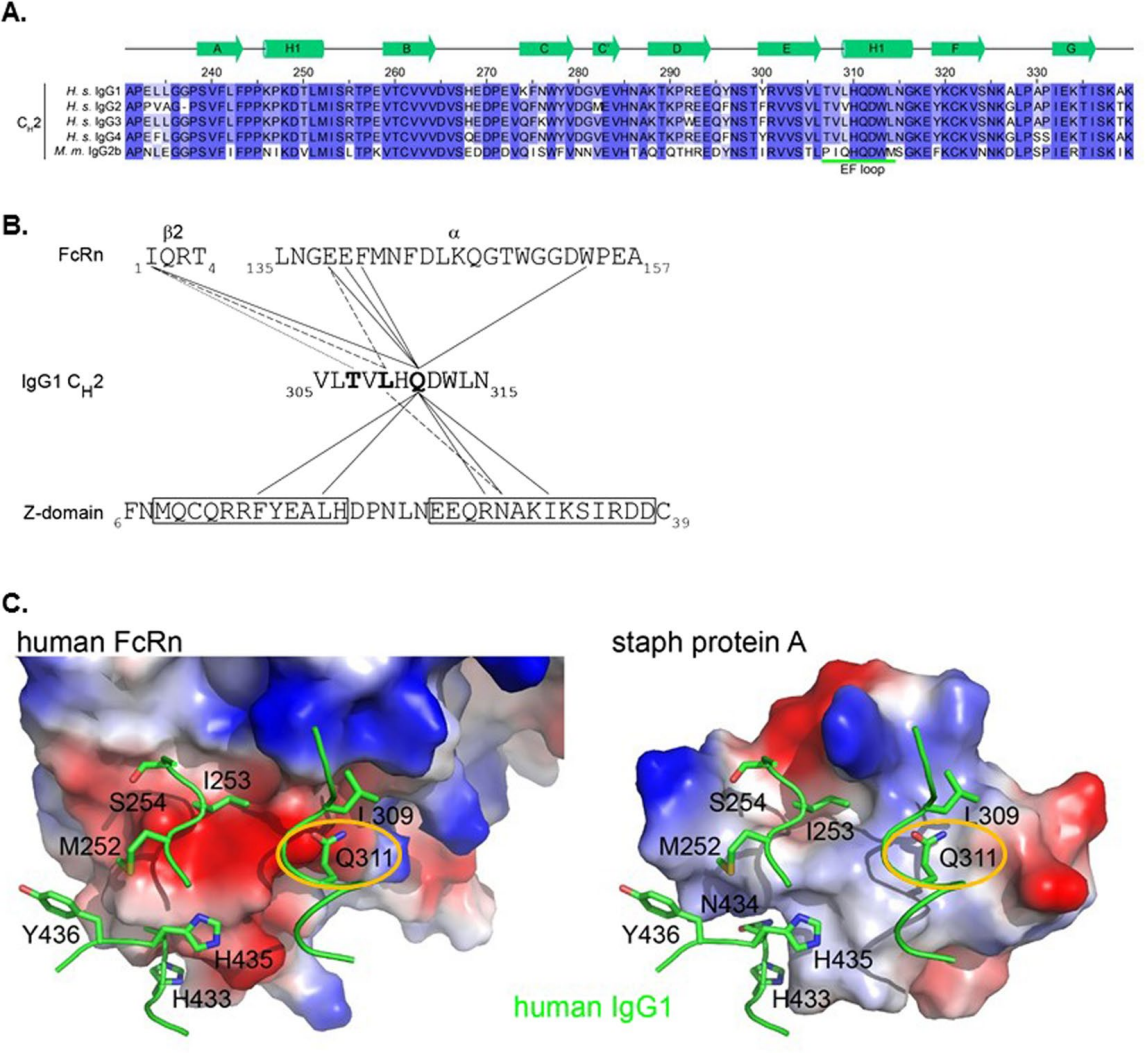
Rationale for Mutations. (**A**) Sequence alignment of the CH2 domains of human IgG and mouse IgG2b. Secondary structures are based on those from PDB ID 1L6X and are shown in green above the alignments. The EF loop, which interacts with Z-doman and FcRn is underlined in green. Residue numbering follows the EU nomenclature. (**B**) Interactions of T307, L309, and Q311 with FcRn or Z-domain. T307, L309, and Q311 (bold) reside in the CH2 domain of the Fc. Each makes side-chain interactions with residues in FcRn (based on PDB ID 4N0U) and with the Z34C peptide, which is a disulfide-bonded two helix bundle derived from Z-domain (from PDB ID 1L6X) based on cutoff distance of 5 Å. T307 interacts with I1 from the β2 microglobulin of FcRn. L309 and Q311R are responsible for interactions with both FcRn and Z-domain (dashed and solid lines for L309 and Q311, respectively). (**C**) Comparison of the electrostatic surface encountered by the Fc when bound to human FcRn (left) or Z-domain (right). IgG residues are shown in green. Q311 (circled) interacts with different surfaces on FcRn vs. Z-domain.

Both FcRn and the protein A-derived Z-domain bind to Fc at the interface between the C_H_2 and C_H_3 domains, contacting many of the same residues on the Fc. We sought to identify sites in the C_H_2 domain which could be mutated to disrupt binding to protein A while leaving the ability of the Fc to bind FcRn unaffected. Since mouse IgG2 binds to Z-domain weaker than human IgG1 while both bind to FcRn^17^, we identified sites in the Z-domain binding interface of human IgG1 Fc which are not conserved in mouse IgG2 (Fig. 1A). Positions 307, 308 and 309 differ between human and mouse IgG, but the difference at position 308 is a change to a similar amino acid: V308I. We reasoned that mutating the human IgG1 to the equivalent mouse IgG2 residues at these sites: T307P, L309Q, may decrease protein A binding while having little effect on FcRn interaction.

We also identified Q311, which interacts with F14, L18, R28, N29 and I32 of Z-domain mostly through hydrophobic interactions (Fig. 1B). Conversely, Q311 interacts with a mostly acidic surface of FcRn (Fig. 1B,C), comprised of E138 and E139 of the α-subunit. We thus reasoned that mutating Q311 could differentially affect binding to Z-domain and FcRn.

### Three mutations selectively disrupt protein A binding

**Figure 2:**
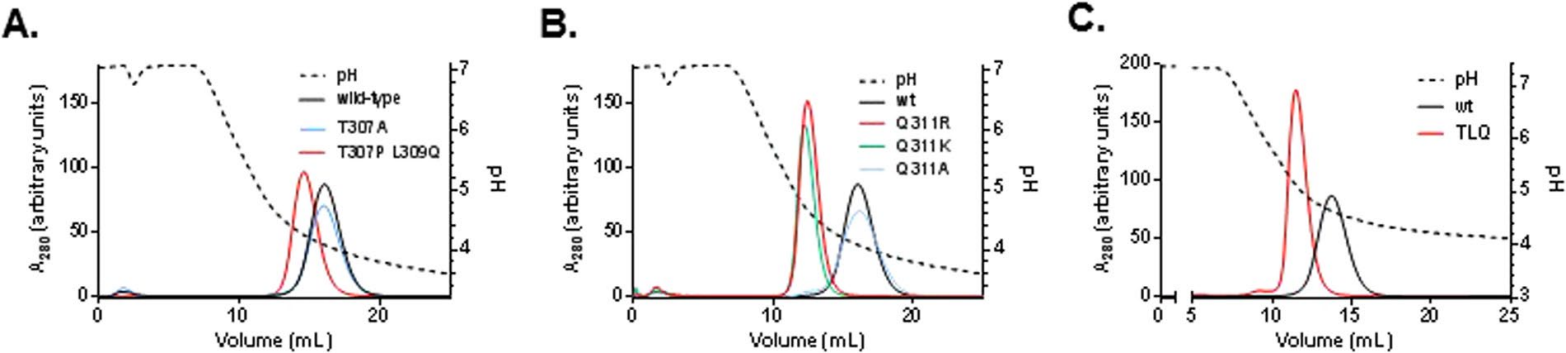
Mutations disrupt Z-domain interaction. IgG1 variants were eluted from a column comprised of Z-domain using a 30 mL gradient from pH 7.2 to pH 3.0 (dotted line). Variants are indicated on the charts. Absorbance at 280 nm was monitored during elution (solid lines).

We tested the extent to which mutations of T307, L309, or Q311 could disrupt binding to Z-domain. Each parental mAb was eluted from a mAbSelect SuRe column (GE Healthcare) and gradient from 1 × PBS pH 7.2 to 50 mM citrate pH 3.5. Wild type human IgG1 eluted from protein A resin at pH 4.09 (Fig. 2A). Whereas mutation of T307A had no effect on protein A binding, mutation of T307P, Q309L resulted in a modest decrease in binding to protein A, causing this mAb to elute at pH 4.48. Additional weakening effect on protein A binding could be achieved by mutation of Q311K or Q311R, but not Q311A (Fig. 2B). We reasoned that the mutations T307P, L309Q, and Q311R (which we call “TLQ” throughout) could be combined to further enhance their effects of disrupting interaction with protein A, which indeed was true, as these mutations allowed elution at pH 4.7 (Fig. 2C). These results show several mutations that each modestly decreases binding to protein A resin, allowing a mutant to a) retain some binding to protein A such that it can be captured and purified by this method and b) elute a higher pH than a wild-type human IgG1, potentially allowing separation of mutant parental, BsAb, and wild-type parental based on differential protein A elution.

### FcRn interaction and pharmacokinetics

**Figure 3:**
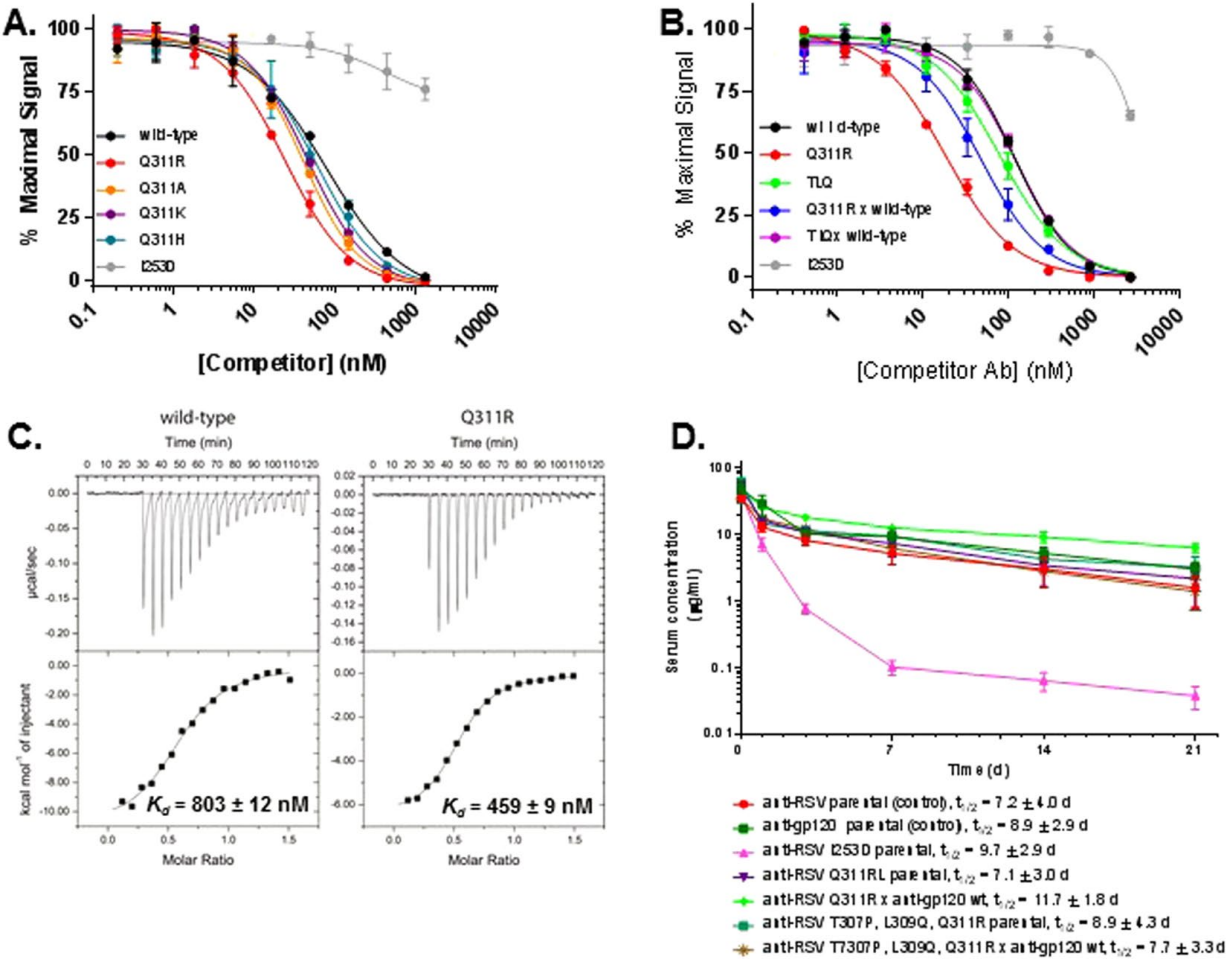
TLQ and Q311R mutants had normal or enhanced FcRn binding. (**A**–**B**) Competition binding of IgG1 variants with wild-type IgG1 for FcRn using AlphaScreen assay. The graph displays % maximum signal plotted vs concentration of competitor. IgG1 wild-type compares the ability of the Ab to compete with itself as a control. (**C**) Isotherms resulting from the binding of FcRn to wild-type IgG1 or IgG1 harboring the Q311R mutation. Top panels show power input vs mole ratio of injectant (IgG) to titrant (FcRn). Bottom panels plot the corresponding binding enthalpy. (**D**) Pharmacokinetic analysis of IgG1variants in Tg32 hemi mice which express the human α-microglobulin subunit of FcRn. The graph displays the concentration of each Ab plotted vs time. Each point represents the mean ± standard error of 4 animals per group.

We asked whether mutations of Q311 which decreased binding to Z-domain could enhance binding to FcRn using a competition-based AlphaScreen method. We used an I253D mutant as a negative control for binding since this mutation is known to disrupt FcRn interaction. None of the mutations at Q311 – R/A/K/H disrupted interaction with FcRn (Fig. 3A). Interestingly, mutation of Q311R led to enhanced ability to bind FcRn. Due to these properties, we used Q311R in combination with T307P and L309Q for further analysis. The TLQ triple mutant bound FcRn with identical ability to wild-type IgG at pH 6.0 (Fig. 3B). BsAb formed from a Q311R or a TLQ mutant paired with a wild-type parental mAb also bound FcRn with identical ability to wild-type IgG1. We asked whether the enhanced ability of the Q311R mutant to compete for FcRn interaction was due to its higher affinity for FcRn. Using isothermal titration calorimetry (ITC), we showed that the Q311R mutant has an ~2-fold higher affinity for FcRn (*K*_*d*_ = 459 nM vs 803 nM for wild-type IgG1) while both Abs displayed the same stoichiometry of 2 FcRn binding sites per Ab (Fig. 3C, Table 1). These results indicated that use of the Q311R or TLQ mutants in combination with a wild-type mAb could result in a BsAb which could be isolated by differential protein A purification and which would have a normal serum-lifetime via FcRn-mediated recycling. A previous study suggested that weaker binding at acidic pH (~pH 6.0) could lead to competition with wild-type IgG1 in the recycling endosome, resulting in increased dissociation and ultimately lysosomal degradation^27^.

**Table 1:** Binding constants and stoichiometry of IgG variants binding to FcRn.

Protein	*K*_*D*_ (μM)*	ΔH (kcal/mol)	−TΔS (kcal/mol)	N*
anti-RSV IgG1 wild-type	0.803 ± 0.05	−8.2 ± 2.2	1.9 ± 0.01	0.57 ± 0.06
anti-RSV IgG1 Q311R	0.459 ± 0.01	−15.2 ± 9.6	2.0 ± 0.02	0.58 ± 0.07

Binding constants and stoichiometry of Z-domain binding to IgG variants. ^*^Average ± standard deviation of 2 or 3 independent measurements.

The I253D mutant Ab, which does not bind FcRn, had a similar terminal half-life as the wild-type parental Abs, but had a much more rapid distribution phase, resulting in a significantly lower area under the curve (AUC) value (Fig. 3D, Table 2). Both the homodimeric parental Abs harboring either Q311R or TLQ had half-lives at least as long as the wild-type mAb (~7 and 9 days, respectively). Unsurprisingly, the mutations also had little effect on serum half-life when paired as a BsAb with a wild-type mAb. The Q311R x wild-type and TLQ x wild-type BsAbs had serum half-lives of 11 and 5 days, respectively. These results are consistent with the *in vitro* FcRn binding analysis and in total show that the mutations allow differential protein A-based purification of BsAb resulting in a molecule which retains normal serum half-life. Other groups have exploited mutation of H435R and Y435F to disrupt protein A interaction and allow differential protein A purification^30,33^. Interestingly, mutation of H435R in human IgG1 was reported to decrease the serum half-life of the Ab^27^. We measured the half-life of human IgG1 harboring either the H435R mutation or the H435R, Y436F mutations in TG32 hemi mice, which express human FcRn (Supplementary Fig. S1, Table 3). The measurements show that the H435R and H435R, Y436F mutants had slightly longer half-lives than a wild-type and TLQ Abs, although they displayed faster initial clearance, having C_max_ values approximately half that of the wild-type or TLQ Abs.

**Table 2:** Pharmacokinetic properties of IgG variants.

Variant	t_1/2_* (d)	C_max_* (μg/mL)	C_last_* (μg/mL)	AUC_last_ (day*μg/mL)	AUC_∞_ (day*μg/mL)
anti-human RSV	7.2 ± 4.0	35.0 ± 3.4	1.6 ± 1.4	117.0 ± 43.6	138.4 ± 61.9
anti-gp120	8.9 ± 2.9	46.2 ± 23.4	3.0 ± 1.1	195.3 ± 9.7	233.7 ± 21.2
anti-RSV I253D	9.7 ± 2.9	39.5 ± 8.2	0.04 ± 0.02	34.6 ± 8.3	35.2 ± 8.4
anti-RSV Q311R	7.1 ± 3.0	47.8 ± 21.9	2.2 ± 2.4	153.1 ± 65.0	182.6 ± 102.3
anti-RSV Q311R x anti-gp120	11.7 ± 1.8	53.2 ± 26.9	5.4 ± 0.8	259.2 ± 13.2	351.3 ± 14.5
anti-RSV T307P, L309Q, Q311R	8.9 ± 4.3	59.9 ± 18.4	3.2 ± 2.4	180.1 ± 42.1	231.3 ± 96.8
anti-RSV T307P, L309Q, Q311R x anti-gp120	7.7 ± 3.3	34.2 ± 4.5	1.4 ± 1.4	138.2 ± 18.6	158.4 ± 22.6
anti-RSV H435R	12.1 ± 3.7	17.8 ± 2.5	1.6 ± 0.8	189.9 ± 44.8	220.1 ± 56.4
anti-RSV H435R, Y436F	13.0 ± 3.2	17.1 ± 6.6	1.5 ± 0.6	181.9 ± 42.1	211.6 ± 56.4

*Abbreviations: t_1/2_: elimination half-life, C_max_: maximum drug concentration, C_last_: drug concentration at last time point, AUC: area under the concentration versus time curve from either point 0 to infinity (AUC_∞_) or to the last time point with quantifiable concentration (AUC_last_).

**Table 3:** Analysis of differential scanning calorimetry data.

Variant	C_H_2 and Fab T_m_ (^°^C)	C_H_2 and Fab ΔH (cal/mol)	C_H_3 T_m_ (°C)	C_H_3 ΔH (cal/mol)
anti-TNFα	70.97 ± 0.01*	6.78 ± 0.02 × 10^5^*	81.75 ± 0.03	1.45 ± 0.02 × 10^5^
anti-TNFα Q311R	71.55 ± 0.01 72.65 ± 0.03	5.33 ± 0.14 × 10^5^ 1.12 ± 0.13 × 10^5^	81.66 ± 0.03	1.65 ± 0.02 × 10^5^
anti-TNFα T307P, L309Q, Q311R	71.55 ± 0.01*	6.81 ± 0.03 × 10^5*^	81.55 ± 0.04	1.61 ± 0.03 × 10^5^

*CH2 and Fab regions could not be deconvoluted.

### Fcγ receptor interaction

Mutation of Q311R or TLQ (parental homodimer mAb or in a BsAb) had no effect on the ability of the Ab to interact with Fcγ receptors *in vitro*, based on a one-way ANOVA test (Supplementary Fig. S2). This result was expected since the mutations exist along the C_H_2-C_H_3 interface, which is responsible for binding FcRn, whereas other Fcγ receptors bind to the C_H_2-hinge interface. The specificity of the effect of the mutations also suggests that they do not perturb the overall structure of the Fc.

### Thermal stability

**Figure 4:**
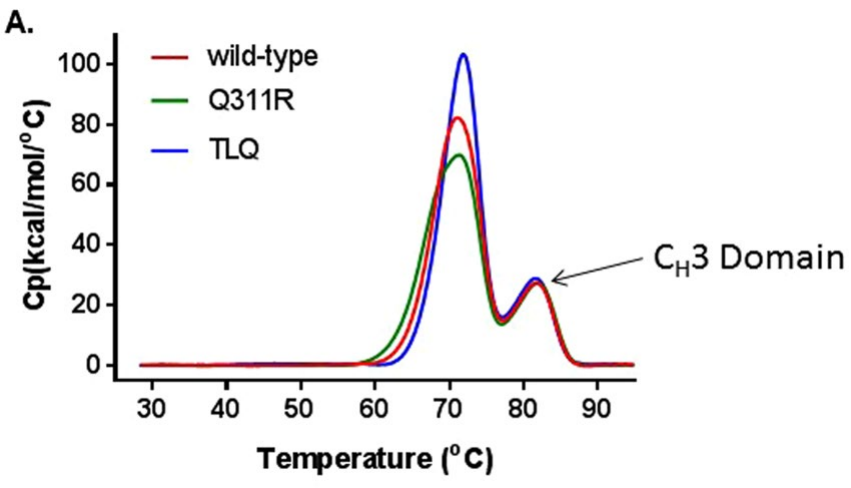
TLQ mutations have no effect on thermal stability of a mAb. Comparison of the thermal unfolding of a wild-type anti-TNFα IgG1 to the same Ab harboring either the TLQ or Q311R mutations, measured by differential scanning calorimetry. Specific heat (Cp) is plotted vs temperature. The transition of the C_H_3 domain is indicated. Data represents the average of two independent experiments.

Mutation of M252Y, S254T, T256E, termed “YTE” in the C_H_2 domain was shown to enhance FcRn binding but led to a decreased melting temperature for the C_H_2 domain, and this was interpreted as a decrease in structural stability of the molecule^34^. This led us to ask whether mutations in the C_H_2-C_H_3 interface would destabilize the Fc region in general. To determine whether the TLQ mutations had a global destabilizing effect on the Fc, we compared the thermal unfolding profiles of the mutants to a wild-type human IgG1. Comparison of the T_m_ values of mutants and wild-type IgG showed the mutations do no perturb the thermal stability of the mAb, since the wild-type, Q311R mutant and the TLQ mutant had CH2/Fab T_m_ between 70–72 °C and a T_m_ for the CH3 domain ~82 °C (Fig. 4, Table 3). Together, these results indicate that the effects of the Q311R and TLQ mutations are localized to protein A and FcRn interaction.

### TLQ mutations allow rapid purification of BsAb from parental mAbs

#### From purified parental mAbs

**Figure 5:**
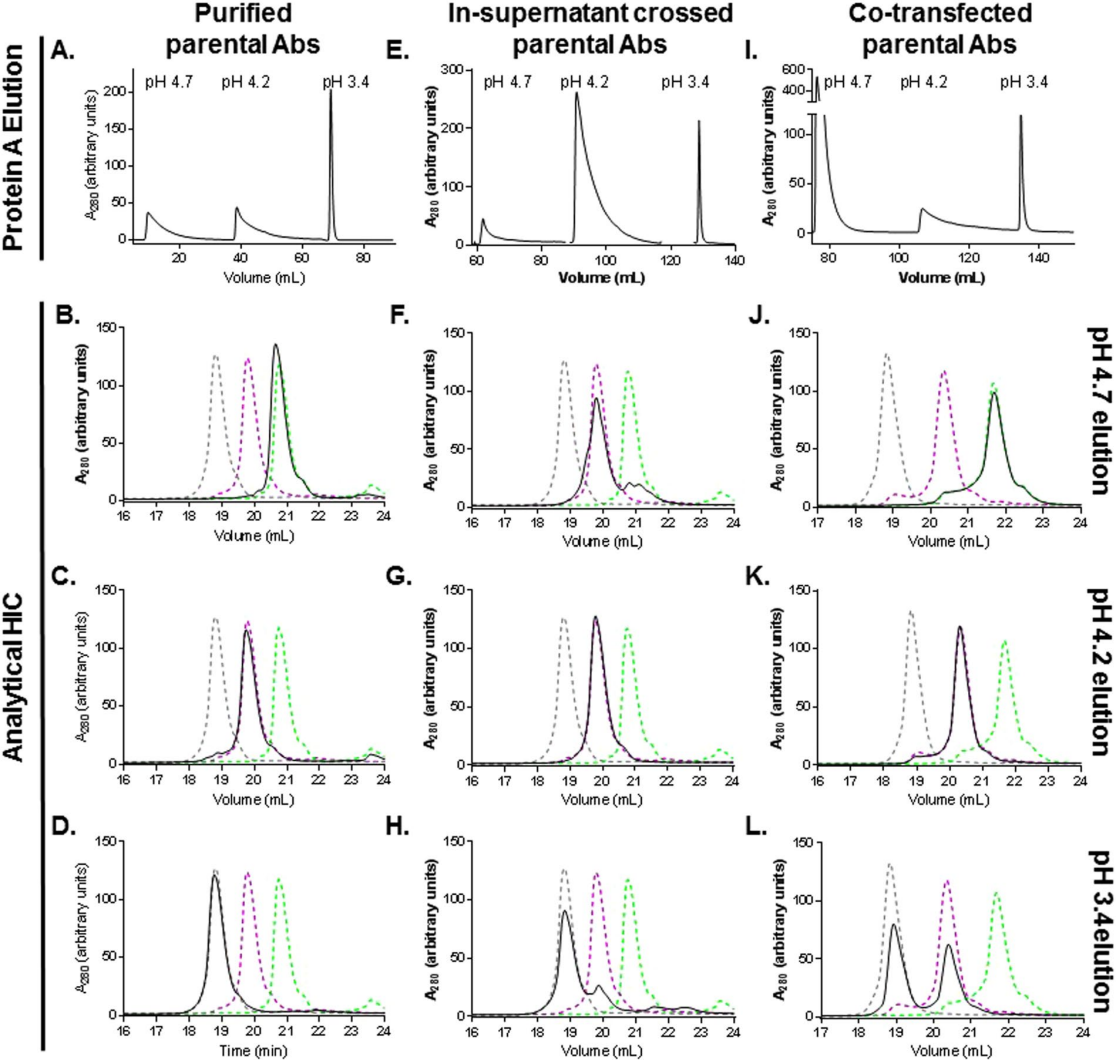
TLQ mutations allow rapid isolation of BsAb. (**A**,**E**,**I**) Differential protein A purification of Ab mixtures using pH step gradients. Absorbance at 280 nm is monitored vs elution volume (mL). (**B**–**D**, **F**–**H**, **J**–**L**) Analytical hydrophobic interaction chromatography analysis of elution peaks from (**A**,**E**, **I**). (**A**–**D**) Purification of a BsAb made by cFAE of purified parental Abs. The TLQ mutant contained an F405L mutation while the “wild-type” molecule contained the K409R mutation. Elution from protein A affinity column monitored by absorbance at 280 nm (**A**). The pH of each elution step is indicated. (**B**–**D**) Protein A affinity elution peaks at pH 4.7, pH 4.2, and pH 3. were analyzed by HIC and are shown in black solid lines. The elution traces of the wild-type parental Ab (black dashed line), the TLQ parental Ab (green dashed line) and the BsAb (purple dashed line) are shown as references. (**E**–**H**) Purification of TLQ BsAb from in-supernatant cFAE. (**E**) Elution from protein A affinity column. Protein A affinity chromatography elutions at pH 4.7 (**F**), pH 4.2 (**G**), pH 3.4 (**H**) were analyzed by HIC. (**I**–**L**) Purification of T307P, L309Q, Q311R BsAb from co-transfected parental mAbs. The TLQ Ab and the wild-type Ab contained no additional mutations for pairing. (**I**) Elution from protein A column monitored by absorbance at 280 nm. Protein A elutions at pH 4.7 (**J**), pH 4.2 (**K**), pH 3.4 (**L**) were analyzed by HIC.

We then focused on the TLQ mutations, which would be paired with a wild-type mAb in BsAb molecules. We asked whether the elution of the wild-type and TLQ mutant parental Abs could be achieved using a step pH gradient to elute from protein A affinity resin. Note that although pH 4.1 was sufficient to elute the wild-type parental Ab (Fig. 2), elution at pH 3.4 resulted in a sharper peak which required less volume for collection. Two IgG1 parental Abs lacking the TLQ mutations eluted as sharp peaks only in the pH 3.4 elution while parental Abs harboring the TLQ mutations eluted almost entirely in the pH 4.7 step. (Supplementary Fig. S3). The TLQ parental mAb also contained an F405L mutation while the wild-type parental mAb contained a complementary K409R mutation, as described previously for cFAE reactions^14^. The approximately equimolar mixture of the TLQ F405L parental, wild-type K409R parental, and the TLQ x wild-type bispecific Abs were purified by differential protein A affinity chromatography using three step segments at pH 4.7, pH 4.2, and pH 3.4, resulting in a distinct elution peak at each pH, (Fig. 5A). The elution peaks were pooled and analyzed by hydrophobic interaction chromatography (HIC) to quantify the population of each species in each elution step. The mutant and wild-type parental mAbs were chosen to contain Fab regions with distinct isoelectric points and hydrophobicity, allowing them to be separated by HIC (Supplementary Figure S3C, Fig. 5B–D). The HIC peaks could then be quantified using absorbance at 280 nm to determine the relative amounts of each molecule in each elution. The high pH elution contained exclusively TLQ parental mAb while the pH 3.4 elution contained > 95% wild-type parental mAb (Table 4). Elution peaks were also analyzed by SDS-PAGE to confirm protein integrity (Supplementary Fig. S3D). Importantly, after starting from an equimolar mixture of parentals and BsAB, the intermediate pH elution contained ~94% pure BsAb, an approximately 3-fold enrichment of BsAb, with >95% recovery.

**Table 4:** Quantitation of BsAb purity starting from a mixture with purified parental Abs.

Elution pH	% TLQ parental	% BsAb	% wt parental
4.7	>99	N.D*	N.D.
4.2	N.D.	94	6
3.4	N.D.	3	97

*N.D. = not detected.

#### From in-supernatant crossed parental mAbs

Current large-scale cFAE protocols to generate BsAb using DuoBody technology require that parental mAb titers in culture supernatant be precisely determined, such that parental mAbs are mixed in a 1:1 molar ratio to generate BsAb. However, cFAE reactions rarely achieve 100% completion and residual amounts of bivalent parental mAb remain. We asked whether the TLQ mutations could allow isolation of BsAb from in-supernatant crossed parental Abs in the same way as above, except that the cFAE reaction was done by mixing culture supernatants from each of the two parental Abs in volumes to result in an equimolar mixture of the two parentals. This mixture was then subjected to cFAE and then loaded onto a protein A affinity column. Similar to the experiment starting from purified parental Abs, the multi-step pH gradient resulted in a distinct peak at each elution pH (Fig. 5E). HIC analysis revealed that the pH 4.2 elution contained >99% BsAb, although the pH 4.7 and pH 3.4 elutions contained significant populations of BsAb (79% and 21%, respectively) (Fig. 5F–H, Table 5). Thus, the yield of BsAb recovered by differential protein A purification was ~90% of the total BsAb. Integration of the absorbance peaks from the purification revealed that the pH 4.7, 4.2, and 3.4 elutions represented 10, 82, and 21.2% of the total eluted population (Fig. 5E). Based on these populations and quantitation of HIC peaks (Fig. 5F–H), we could calculate both the extent of BsAb formation and yield from differential protein A elution. The extent of BsAb formation from in-supernatant cFAE was ~91% and the yield was ~90% of the total BsAb produced.

**Table 5:** Quantitation of BsAb purity starting from in-supernatant crossed parental mAbs.

Elution pH	% TLQ parental Ab	% BsAb	% wt parental Ab
4.7	21.4	78.6	N.D.
4.2	N.D.	>99	N.D.
3.4	N.D.	21.2	78.8

*N.D. = not detected.

#### From co-transfected parental mAbs

We asked whether the TLQ mutations could be used to isolate BsAb from co-transfected parental mAbs lacking mutations to enhance BsAb formation (i.e. F405L/K409R). To minimize the potential Ab species which could be generated by light chain mispairing, we used a set of parental mAbs sharing a common light chain, and whose V_H_ domains had been charge-engineered to allow separation by HIC. As for the applications of differential protein A elution descried above, the purification resulted in a distinct peak at each elution pH (Fig. 5I). HIC analysis revealed that the pH 4.2 elution contained >95% pure BsAb while the parental Abs were efficiently eluted at either pH 4.7 or pH 3.4 (Fig. 5J–L, Table 6). Interestingly, the peak from the pH 4.7 elution represented about 74% of the total population, whereas the peak from the pH 4.2 elution represented ~17% and the 3.4 elution peak contained only ~9% of the population, suggesting either that one parental Ab expressed much more highly than other or that either the BsAb or wild-type parental Ab was eluting at a higher pH. We calculated the total population of BsAb in the co-transfected supernatant as 21%. While the purity of BsAb isolated from pH 4.2 elution was high (~98%), total yields of BsAb were slightly lower than expected due to the significantly different expression levels of the two parental mAbs when co-transfected (~300 mg/L for TLQ parental mAb vs ~35 mg/L for wild-type parental mAb). This difference in co-expression titers is likely specific to each mAb, and the example presented is a relatively extreme case. Despite this ~10-fold difference in parental mAb expression levels, the pH 4.2 elution contained ~ 90% of the total BsAb produced, despite its low abundance in the total population of protein (in which it accounted for only ~21% of the total species) from co-transfected material.

**Table 6:** Quantitation of BsAb purity starting from co-transfected parental mAbs sharing a common light chain.

Elution pH	% TLQ parental Ab	% BsAb	% wt parental Ab
4.7	2.9	89.6	7.4
4.2	1.3	96.8	1.9
3.4	N.D.	47.6	52.4

*N.D. = not detected.

## Discussion

BsAbs provide significant clinical advantage over mono-specific mAbs for dual antigen recognition and effector cell redirection. For example, an anti-EGFR x anti-cMet BsAb is able to simultaneously bind both antigens which are highly expressed on non-small cell lung carcinoma (NSCLC) cells^35^. The BsAb has more potent anti-tumor activity than combination therapy of the two parental mAbs and shows efficacy in tumors which are resistant to EGFR monotherapy and was amenable to combination therapy with tyrosine kinase inhibitors. In another example, an anti-CD33 x anti-CD3 BsAb could successfully redirect cytotoxic T cells to acute myeloid leukemia (AML) cells leading to efficient killing of residual AML cells which frequently go unaffected by standard therapies and often lead to tumor relapse^36^. BsAbs have the potential to greatly expand the biologic therapeutic arsenal against disease. However, generation of therapeutic BsAb is time-consuming, and expensive. Downstream methods to purify BsAbs for clinical trials are unique to each molecule. These limitations ultimately contribute to higher costs for patient therapies. Thus, methods to produce BsAb quickly and reliably offers considerable benefit.

Here, we have identified three mutations in the Fc of human IgG1 isotypes which confer decreased binding to protein A while maintaining binding to FcRn. These mutations: T307P, L309Q, and Q311R or “TLQ” allow rapid and reproducible isolation of >95% BsAb from previously purified parental mAbs and in-supernatant crossed parental mAbs containing F405L/K409R mutations or from co-transfected mAbs which share a common light chain but which lack other pairing mutations (Fig. 5). We used a three elution steps at pH 4.6, 4.2, and 3.4 to elute each of the major species present in the mixture. This method requires that the pH of the elution buffers be carefully calibrated to ensure high purity of the BsAb and the type of protein A resin used. In this study we used mAbSelect SuRe resin (GE Healthcare) which is comprised of a protein A derivative with slightly higher affinity than native protein A. The TLQ parental Ab can be eluted at pH 4.6 with little contamination of the BsAb, but at pH values lower than 4.5, the BsAb begins to elute from the column. Likewise, at pH values lower than 4.1, the wild-type parental Ab begins to elute from the column, such that for elution of the BsAb, pH values less than 4.1 should be avoided. To ensure the robustness of the elution pH values, the data presented in this study are representative of at least three independent replicates. The recovery appeared to be independent of the starting purity of BsAb – our data shows that >95% pure BsAb can be recovered from culture supernatant starting from a BsAb population representing only ~21% of total Ab population, with yields of approximately 90%. Interestingly, the Q311R mutation conferred a 2-fold increase in affinity for FcRn while decreasing affinity for protein A (Figs 2–3 **)**. This increase in FcRn binding affinity did not appear to manifest in significant half-life extension *in vivo* although these mutants had half-lives similar to wild-type IgG1 (Fig. 3D).

Mutations which alter protein A binding and allow differential protein A elution of BsAbs have been described. Another group described mutations based on sequence comparison of human IgG1 vs IgG3 which showed that IgG3, which does not bind protein A efficiently, differs from IgG1 by having R435, F436. Human IgG1 mAbs harboring the mutations: H435R and R436F have no ability to bind protein A and allow purification of a BsAb harboring these mutations on only one heavy chain based on analogous methods analogous to those described here^30^. However, H435 is important for FcRn interaction and the presence of R435 in human IgG3 is largely responsible for the short serum half-life of IgG3 since it helps to mediate pH dependent FcRn binding^27,32,37^. Previous work suggested mutation of H435R in human IgG1 led to a serum half-life equivalent to IgG3 and the converse mutation of R435H in IgG3 could recover serum half-life to that of an IgG1 via an FcRn-dependent mechanism^27^. The additional mutation of Y436F may mediate recovery of FcRn interaction, and thus recovery of serum half-life, for the H435R, Y436F mutant, but this has not been shown directly. However, our results suggest that both the H435R and H435R, Y436F mutants have serum half-lives identical to a wild-type Ab (Supplementary Fig. S2). One advantage of this mutation set is that the total disruption in protein A binding caused by the mutations may allow larger pH differences in elution of the parental and BsAb species, leading to higher yields. On the other hand, purification of parental mAbs harboring these mutations will require an alternative resin, such as protein G.

Unexpectedly, although the Q311R mutation described here could enhance FcRn interaction *in vitro* by 2-fold, a mAb harboring this mutation had a half-life similar to that of a wild-type human IgG1 (Fig. 3C,D). Previously described mutations in the C_H_2 domain, namely M252Y/S254T/T256E, or “YTE” were shown to enhance binding to human FcRn *in vitro* by ~10-fold^25,38^. This increase in *in vitro* FcRn binding was correlated to at least 2-fold longer serum half-life for the YTE variant in cynomolgous monkeys and transgenic mice which express human FcRn^38,39^. These results suggest that large increases in binding affinity may be correlated to more modest half-life extension *in vivo* and thus, a 2-fold increase in binding affinity displayed by the Q311R mutant may not result in a significant half-extension. While the YTE mutant was shown to have a significant effect on serum half-life, these mutations were also shown to decrease the melting temperature of the C_H_2 domain and lead to a decrease in affinity for Fc γ receptor IIIA^34,38^, whereas the Q311R mutation or TLQ mutations had no effects on thermal stability of the mAb or its abilities to bind Fc γ receptors RI, RIIa, RIIb, or RIIIa (Fig. 4, Supplementary Fig. 2). These results strongly suggest that the effects of the TLQ mutations are specific to protein A binding and do not mediate allosteric effects on the structure of the IgG. Additionally, in silico analysis suggested low immunogenic potential of the TLQ mutations since the mutations do not introduce any known MHC ligands^40^, although further testing using *ex vivo* methods was not performed.

The results presented here describe a set of mutations: T307P, L309Q, Q311R, or TLQ, which allow single-step purification of a BsAb or heterodimeric Fc molecule to >95% purity. These mutations disrupt protein A interaction while modestly enhancing FcRn binding *in vitro*. mAbs or BsAbs harboring the TLQ or Q311R mutations have serum half-lives at least as long as a wild-type human IgG1. Thus, BsAbs harboring the TLQ mutations are amenable for *in vivo* studies and downstream development.

## Methods

### Proteins

Protein residue numbering followed the EU numbering system. All Abs were expressed in Expi293F cells (ThermoFisher Scientific). For differential protein A purification from purified parental mAbs and from in-supernatant crossed material, variants were generated in an anti-RSV mAb and were paired with a wild-type anti-gp120 mAb. For experiments using cFAE, the TLQ parental mAb also contained an F405L mutation while the wild-type parental mAb contained a complementary K409R mutation. The parental mAbs were purified by protein A affinity chromatography and dialyzed into 1 × PBS. The two parental mAbs were then subjected to cFAE at 1 mg/mL as described^14^. Briefly, 5 mg of each parental were mixed in buffer containing 1 × PBS, 75 mM 2-mercaptoethylamine and incubated at 31 °C for 5 hr followed by extensive dialysis against 1 × PBS. For co-transfection experiments, a set of anti-TNFα and anti-αV integrin sharing a common light chain and whose heavy chains had been charge-engineered to provide analytical separation were used.

### Protein A affinity Purification

#### Determination of elution pH

For each parental mAb harboring the mutations on both arms, 1 mg was loaded onto a 1 mL mAbSelect sure column (GE Healthcare) and eluted at 1 mL/min using a 30 mL gradient from 1 × PBS pH 7.2 to 50 mM citrate pH 3.5. Absorbance at 280 nm and pH were monitored. The pH value at the peak maximum was used to determine the elution pH for preparative experiments.

#### Differential protein A purification from previously purified parental mAbs

A BsAb was mixed in a 1:1:1 molar ratio with the two parental mAbs prior to differential protein A purification. Differential protein A purification was carried out using a 1 mL mAbSelect Sure column (GE Healthcare). The mixture was eluted in 3 steps using buffers containing either 50 mM citrate pH 4.7, pH 4.2, or pH 3.4. Elution fractions were collected and concentrated to >1 mg/mL prior to analysis. The pooled elution peaks from the differential protein A purification were analyzed by hydrophobic interaction chromatography (HIC) using a butyl NPR column (Tosoh Biosciences). Approximately 30 ug of each sample were injected onto the column and eluted using a 0 to 100% gradient of buffers containing 100 mM sodium phosphate pH 6.0, 1.5 M (NH_4_)_2_SO_4_, or 100 mM sodium phosphate.

#### Differential protein A purification from in-supernatant crossed parental mAbs

Parental mAbs containing either Q311R or TLQ in combination with F405L, and mAb containing K409R (F405L and K409R are required for cFAE) were expressed in Expi293F cells and antibody titers were determined (Octet, ForteBio). To produce BsAb, culture supernatant containing equivalent milligram quantities of parental mAbs were mixed as follows: TLQ, F405L + K409R. cFAE reactions were performed at a final protein concentration of 0.2 mg/mL as described above. Following dialysis proteins were applied to a 1 mL mAbSelect Sure column (GE) and eluted using a pH step gradient. TLQ × wild-type BsAb produced from in-supernatant crossed parental mAbs was eluted with 50 mM citrate pH 4.7 for 30 CV followed by 50 mM citrate pH 4.2 for 30 CV followed by 50 mM citrate pH 3.4 for 20 CV. Efficiency of separation was assessed using HIC as described above.

#### Differential protein A purification from co-transfected parental mAbs

Transfections were carried out in Expi293 cells according to the manufacturer’s protocol using a molar ratio of 0.5: 0.5: 3.0 of plasmid for heavy chain 1: heavy chain 2: common light chain. To determine the approximate relative expression levels, separate transfections of parental mAbs were also performed using a 1.0: 3.0 molar ratio of heavy chain: light chain plasmids and titers determined using Surface Bilayer Interferometry (Octet, Forté Bio). Approximately 50 mL of each supernatant were applied to a 1 mL mAbSelect Sure column and eluted using a 3-step pH step gradient of pH 4.7 (or 4.6), 4.2, and 3.4 citrate buffer. Fractions were collected, concentrated and buffer exchanged into 1 × PBS prior to HIC analysis.

### Fc receptor Binding by AlphaScreen

Abs were biotinylated using the SureLINK Chromophoric Biotin Labeling kit (KPL Inc.), according to the manufacturer’s protocol. His-tagged FcRn was purchased from Sino Biological. Assays were performed in 1 × PBS adjusted to pH 6.0, supplemented with 0.05% (w/v) bovine serum albumin (BSA) and 0.01% (w/v) Tween-20. Biotinylated wild-type IgG1 at 1 μg/mL was bound to streptavidin-conjugated donor beads, and His-tagged FcRn at 0.2 μg/mL was bound to a nickel-conjugated acceptor bead. Competitor Abs were prepared at 0.4 mg/mL and were serially diluted by 3-fold for each point. Luminescence between 520–620 nm was recorded using an EnVision plate reader (Perkin Elmer). Data were analyzed using Prism 6.01 software (GraphPad Software, Inc.) and fit using a 4-parameter competition model, as described previously^41^. His-tagged FcγR were purchased from R&D systems. Assays were performed in 1 × PBS pH 7.2, supplemented with 0.05% (w/v) bovine serum albumin (BSA) and 0.01% (w/v) Tween-20. Biotinylated wild-type IgG1 at 1 μg/mL was bound to streptavidin-conjugated donor beads, and His-tagged FcγRs were bound respectively to a nickel-conjugated acceptor bead. Note that for FcγRI, a biotinylated IgG1 containing L234A/L235A mutations, which binds the receptor weaker than wild-type IgG1^42^, was used to increase the signal window. The concentrations of FcγRs used were 200 ng/mL (FcγRI and Fcγ RIIIa), 10 ng/mL (FcγRIIa), or 14 ng/mL (FcγRIIb). Competitor Abs were prepared at 0.4 mg/mL and were serially diluted by 3-fold for each point.

### Pharmacokinetic analysis

For the pharmacokinetic (PK) studies, transgenic Tg32 hemizygous mice expressing the human α-microglobulin subunit of FcRn (Supplementary Fig. S2) were used to better predict serum half-life in humans. Mice were injected with test Abs intravenously via tail vein at a dose of 2 mg/kg into 4 animals per group. Time points were taken at 1 h, 1 d, 3 d, 7 d, 14 d and 21 d. Serial retro-orbital bleeds were obtained from CO_2_-anesthesized mice at the indicated time points and terminal bleeds were taken by cardiac puncture. After 30 min at room temperature, blood samples were centrifuged 3,000 × g for 15 min and serum collected for analyses. The PK study was approved by the Institutional Animal Care and Use Committee at Janssen Research & Development, LLC. All experiments were performed in complicance with the guidelines of the committee.

For detection of the test Abs in mouse sera, an electrochemiluminescent immunoassay was used. Streptavidin Gold multi-array 96-well plates (Meso Scale Discovery) were coated overnight with 50 µL/well of 3 µg/mL Biotin-F(ab′)_2_ anti-human IgG, Fc fragment specific (Jackson Immunoresearch cat. # 109-066-008) in Starting Block (Thermo); then washed in Tris-buffered saline with Tween 20 (TBST). Sera samples were diluted in 5% CD-1 mouse serum in Starting Block (1:20, then serial 2-fold dilutions), incubated on plates for 2 h and washed. Ru^2+^-labeled anti-human IgG F(ab′)_2_ (prepared from Jackson 109-006-097) in 1% BSA-TBST was added and incubated on plates for 1.5 h and washed. Two hundred microliters/well of Read Buffer with surfactant was added and plates were read in a MSD Sector Imager 6000 plate reader. Serum concentrations of the Abs were determined from a standard curve using a 4-parameter non-linear regression program in Prism 6.01 software.

Terminal half-life (t_1/2_) calculations of the elimination phase (β phase) for PK studies were determined using the 1-phase exponential decay model fitted by non-linear regression of natural log concentration versus time using Prism version 6.01 software. The least squares nonlinear decay model was weighted by the inverse of the fitted concentration. Half-life calculations of the elimination phase (β phase) were determined using the formula t_1/2_ = ln2/β, where β is the –slope of the line fitted by the least square regression analysis starting after first dose. The terminal half-life value for an Ab was determined by taking the average of the t_1/2_ values calculated for each animal within the test group.

### Isothermal Titration Calorimetry

ITC experiments were performed at 25 °C using a MicroCal VP-ITC calorimeter. Both titrant and injectant were dialyzed in PBS adjusted to pH 6.0. All titrations were performed analogously, having 10 μM FcRn as the titrant and 70 μM of IgG as the injectant. Each 14 μL injection of IgG spanned 32 s, with 5 min spacing between injections. Data were analyzed using Origin software using a 1:1 binding model.

### Differential Scanning Calorimetry

Differential scanning calorimetry (DSC) was used to determine the T_m_ and enthalpies of unfolding for a wild-type, Q311R, and a TLQ mAb. Samples were diluted to 1 mg/mL in 1 × PBS pH 7.2. Samples were equilibrated to 25 °C for 15 min prior to temperature ramping from 25–95 °C at a rate of 1 °C/min. Data was analyzed using Origin software.

## Electronic supplementary material

Supplementary Information
