# Supplementary material for: Rapid Purification of Human Bispecific Antibodies via Selective Modulation of Protein A Binding
Source: Sci Rep. 2017 Nov 14;7:15521. doi: 10.1038/s41598-017-15748-0 (PMC5686111; doi:10.1038/s41598-017-15748-0)
Supplement: Supplementary file 1 — Supplementary Information [file 41598_2017_15748_MOESM1_ESM.doc]

**Rapid Purification of Human Bispecific Antibodies via Selective Modulation of Protein A Binding**

**Adam Zwolak^1^, Catherine Leettola^1^, Susan H. Tam^1^, Dennis R. Goulet^2^, Mehabaw Derebe^1^, Jose R. Pardinas^1^, Songmao Zheng^3^, Rose Decker^4^, Eva Emmell^4^, Mark Chiu^1*^**

^1^Biologics Research, Janssen Research & Development, LLC, Spring House, PA 19477, USA

^2^Department of Medicinal Chemistry, University of Washington, Seattle, WA 98195, USA

^3^Biologics Development Sciences, Janssen Research & Development, LLC, Spring House, PA 19477, USA

^4^Biologics Toxicology, Janssen Research & Development, LLC, Spring House, PA 19477, USA

*mchiu@its.jnj.com

**Supplementary Information**

**
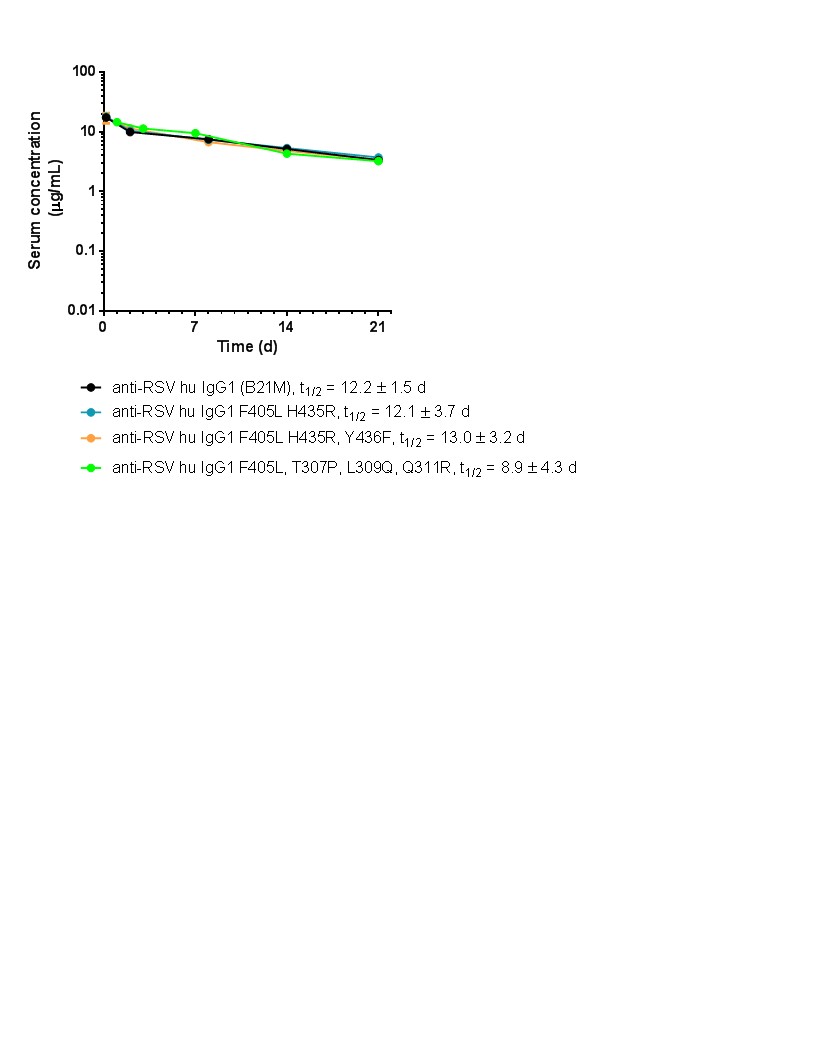
**

**Supplementary Figure S1. Pharmacokinetic analysis of H435R, Y436F mutants.** Pharmacokinetic analysis of IgG1variants in TG32 hemi mice, which express the human α-microglobulin subunit of FcRn. The graph displays the concentration of each Ab plotted vs time. Each point represents the mean ± standard error of 4 animals per group.


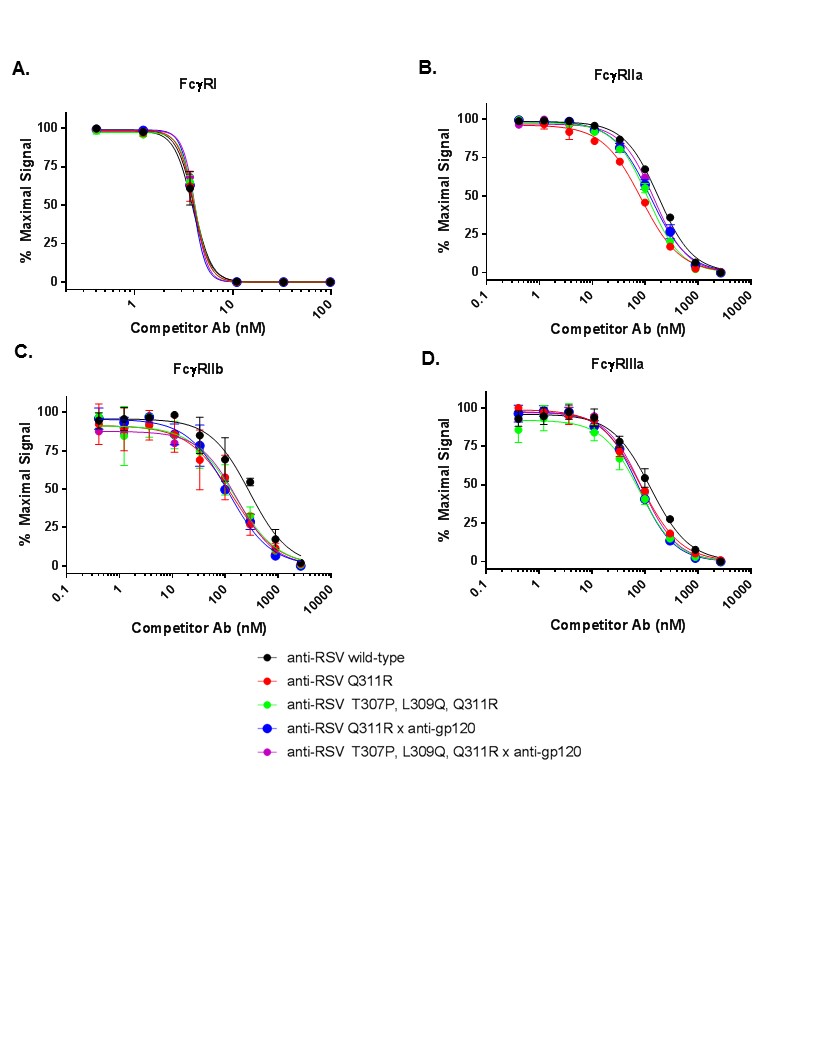


**Supplementary Figure S2. Analysis of FcγR interaction.** Competition binding of human IgG1 variants with wild-type IgG1 for FcγRI (A), FcγRIIa (B), FcγRIIb (C), and FcγRIIIa (D) using AlphaScreen assay. The graph displays % maximum signal plotted vs concentration of competitor. IgG1 variants are described in the figure. Human IgG1 wild-type compares the ability of the Ab to compete with itself as a control.


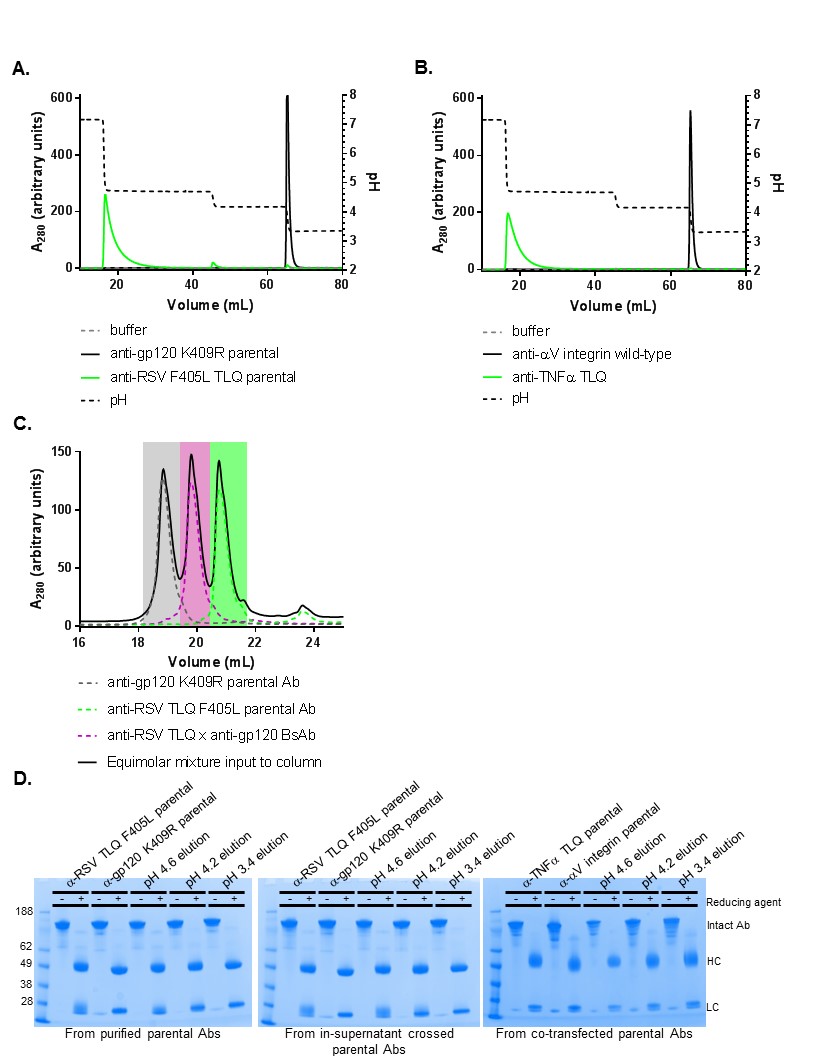


**Supplementary Figure S3. Differential protein A elution of parental Abs.** Wild-type parental IgG1 Abs (black) or TLQ mutant parental Abs (green) were eluted from a protein A affinity column using a step gradient at pH 4.7, 4.2, and 3.4. The anti-gp120, anti-RSV pair (A) represent the parental Abs shown in panels A and E of Figure 5. These parental Abs contain the F405L/K409R pair of complementary mutations for cFAE reactions. The anti-αV integrin, anti-TNFα pair (B) represent the parental Abs shown in panel I of Figure 5. These parental Abs contained no additional mutations for HC-HC pairing. The pH of each step is shown on the right y-axis while absorbance is plotted on the left y-axis vs elution volume. (C) Analytical HIC analysis of the purified parental Abs, the BsAb, and the equimolar mixture of all three species which was applied to the protein A affinity column in Fig. 5A. The anti-RSV TLQ F405L parental Ab eluted at approximately 21 mL, the anti-gp120 K409R parental Ab eluted at approximately 19 mL while the BsAb eluted at approximately 20 mL. The cutoff volumes used to quantify each species are indicated by the shaded areas and are color-coded to each species. (D) SDS-PAGE analysis of pH step elutions from Figure 5. Left panel corresponds to Figure 5A, center panel corresponds to Figure 5E, and right panel corresponds to Figure 5I. Species are indicated in the Figure.
